# Case Report: Surgical Reconstruction of a Massive Thoracic Wall Defect After the Resection of an Undifferentiated Radiation-Induced Sarcoma of the Breast

**DOI:** 10.3389/fsurg.2021.773313

**Published:** 2021-11-11

**Authors:** Marco Rastrelli, Claudia Di Prata, Roberto Marconato, Paolo Del Fiore, Elisa Granziera, Antonella Brunello, Vincenzo Vindigni, Andrea Zuin, Marta Sbaraglia, Saveria Tropea, Simone Mocellin

**Affiliations:** ^1^Soft-Tissue, Peritoneum, and Melanoma Surgical Oncology Unit, Veneto Institute of Oncology IOV-IRCCS, Padua, Italy; ^2^Department of Surgery Oncology and Gastroenterology, University of Padova, Padua, Italy; ^3^Anesthesiology Unit, Veneto Institute of Oncology IOV-IRCCS, Padua, Italy; ^4^Department of Oncology, Oncology 1, Veneto Institute of Oncology IOV-IRCCS, Padua, Italy; ^5^Clinic of Plastic Surgery, Department of Neuroscience, Padua University Hospital, University of Padua, Padua, Italy; ^6^Thoracic Surgery Unit, Department of Cardiologic, Thoracic, and Vascular Sciences, University of Padua, Padua, Italy; ^7^Department of Pathology, Azienda Ospedale-Università Padova, Padua, Italy; ^8^Department of Medicine, University of Padua School of Medicine, Padua, Italy

**Keywords:** radiation induced sarcoma, chest wall sarcoma, adjuvant radiation therapy, breast, breast cancer

## Abstract

A 54-year-old lady was referred to our institute because of a massive thoracic neoplasm arising from the thoracic wall which infiltrated and dislocated the left breast. Twenty years before, the patient had undergone a quadrantectomy with axillary dissection for an infiltrating ductal carcinoma of the left breast, followed by adjuvant radiotherapy and chemotherapy. A true-cut biopsy of the mass showed a low differentiated malignant neoplasm with spindle-shaped cells. The patient underwent a total-body CT scan which showed a 16 × 15 × 10 cm largely necrotic mass with irregular and undefined margins, with little homolateral round-shaped cervical and mesenteric lymph nodes but no distant metastases. After a multidisciplinary discussion, we proposed surgery as the first therapeutic option. The planned treatment was a wide excision of the mass with the underlying ribs (II-VI) followed by the reconstruction of the thoracic wall using titanium bars covered by the acellular porcine dermis, latissimus dorsi flap, and finally, skin grafts from the thighs.

## Introduction

The association between adjuvant radiotherapy for breast cancer and the late development of soft tissue sarcoma, although rare, is well-established. Iatrogenic radio-associated sarcoma (RIS) represent 3% of all soft tissue sarcomas and among these, the most frequent are cutaneous angiosarcoma and undifferentiated pleomorphic sarcoma. Surgery, if feasible, still represents the most effective treatment.

## Case Presentation

**Figure 1:**
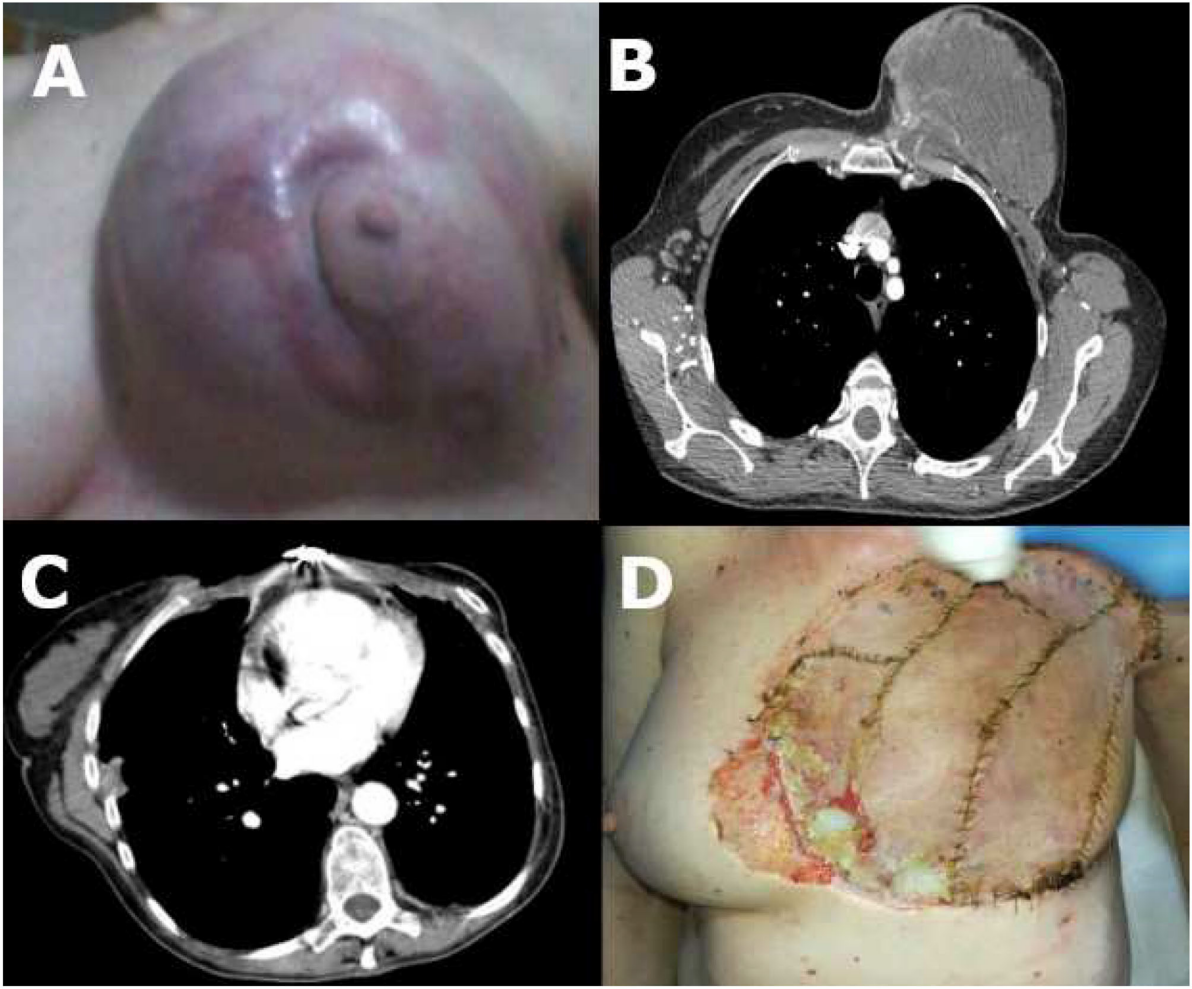
**(A)** Chest wall sarcoma before surgery; **(B)** CT scan before surgery; **(C)** CT scan after surgery; **(D)** Chest wall sarcoma after surgery with small ulceration (low-right quadrant).

A 54 year-old lady was referred to our institute because of a massive thoracic neoplasm arising from the thoracic wall which infiltrated and dislocated the left breast. The mass measured 16 × 15 × 10 cm and appeared solid and firmly anchored to the underlying thoracic wall; it was moderately painful, and the surrounding skin appeared edematous, but not ulcerated (Figure 1A).

The self-discovered mass showed itself 5 months before as a solid, firm nodule in the left breast. The nodule grew fastly and 1 month after the initial discovery, an MRI documented a 10 cm mass in the left breast, iper-vascularized and metabolically active on a CT-PET scan.

The patient underwent a total-body CT scan which showed a 16 × 15 × 10 cm largely necrotic mass with irregular and undefined margins, with little homolateral round-shaped cervical and mesenteric lymph nodes but no distant metastases (Figure 1B).

**Figure 2:**
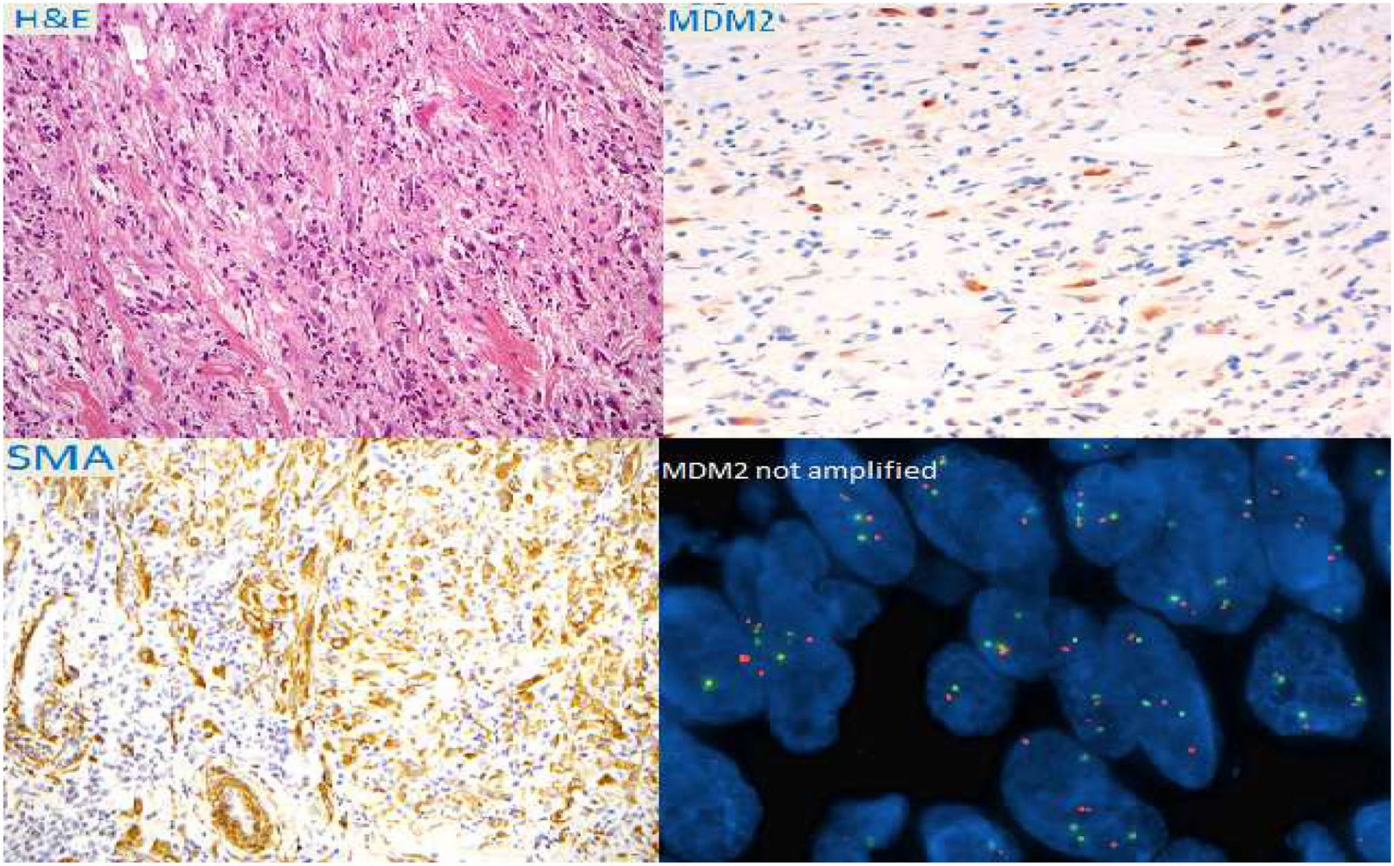
Immuno-histochemical colorations and fluorescence *in situ* hybridization (FISH) of the bioptical specimen.

A true-cut biopsy of the mass showed a low differentiated malignant neoplasm with spindle-shaped cells and 13 mitoses/HPF. The fluorescence *in situ* hybridization (FISH) test excluded the amplification of the MDM2 gene (Figure 2).

Twenty years before, the patient had undergone a quadrantectomy with axillary dissection for an infiltrating ductal carcinoma of the left breast, followed by adjuvant radiotherapy and chemotherapy. She had three children and two abortions. Her father died of colorectal cancer.

The patient used to take 50 mg/bid of Oxycodone for pain relief.

After a multidisciplinary discussion, we proposed surgery as the first therapeutic option. The planned treatment was a wide excision of the mass with the underlying ribs (II-VI) followed by the reconstruction of the thoracic wall using titanium bars covered by the acellular porcine dermis, latissimus dorsi flap, and finally, skin grafts from the thighs. An optimal parietal stabilization with a satisfying pulmonary function is impossible to obtain using only meshes without rigid underlying support, after a very wide chest wall resection, involving the lower portion of the sternum and the anterior arch of five ribs (from the second to the sixth).

During the operation, the pectoralis muscles, the inferior part of the sternum, and the II-VI ribs appeared macroscopically infiltrated by the tumor and were therefore excised. Cutaneous margins were analyzed intraoperatively and resulted free from malignant infiltration.

**Figure 3:**
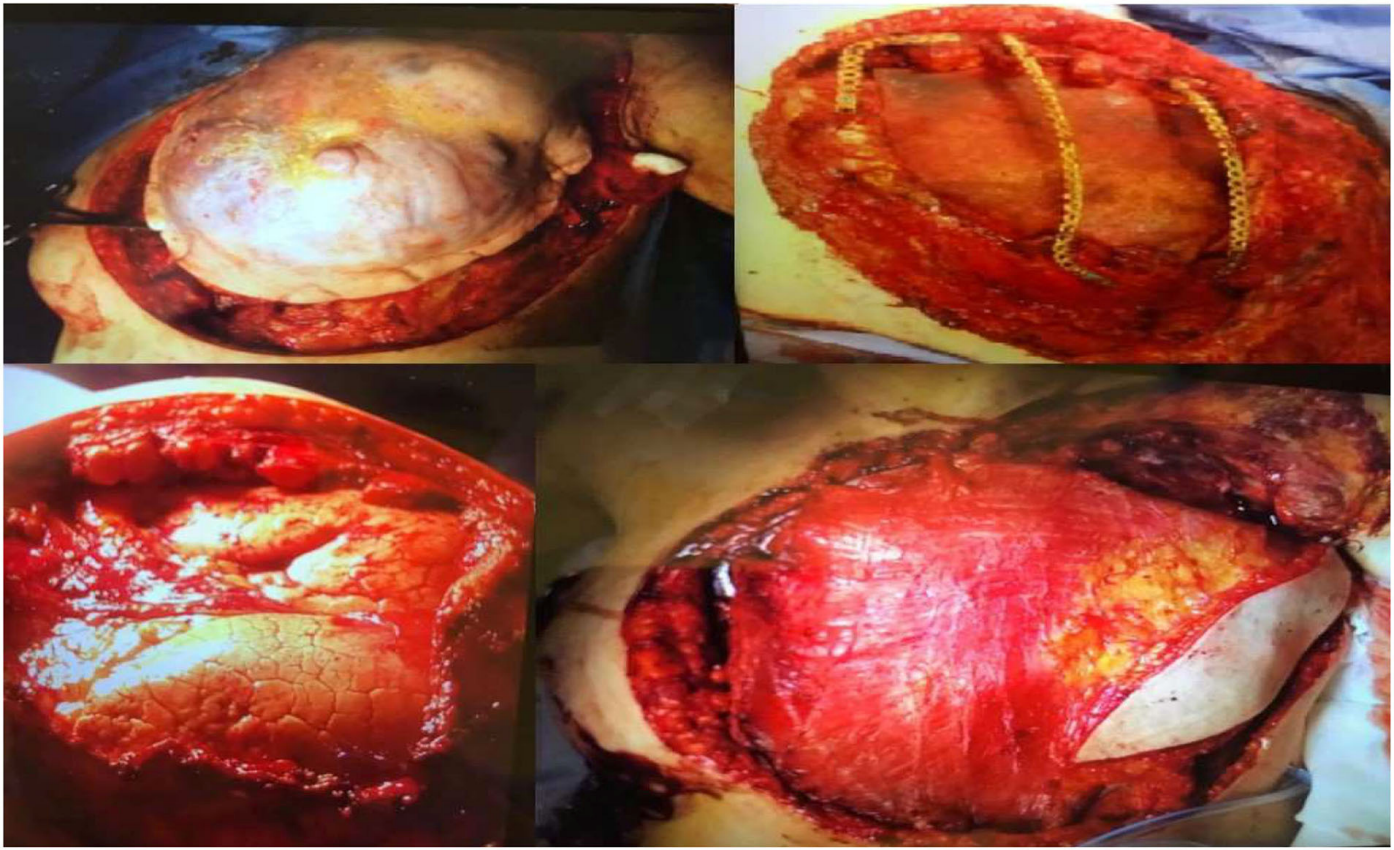
Intraoperative sequences.

The thoracic wall defect was repaired with titanium bars anchored to the remaining portion of the excised ribs and the sternum and enveloped in a double layer of the porcine acellular dermis. Finally, the external layer of the porcine dermis was covered with a latissimus dorsi mio-cutaneous flap and skin grafts from the thighs (Figure 3).

Six drainages were placed: two chest tubes, one drainage above the deep layer of the porcine dermis, one in the donor site of the latissimus dorsi flap, and two under the flap.

After the operation, the patient was transferred to the intensive care unit (ICU) for post-operative monitoring, and 5 days after the operation, she was re-admitted to our ward. During her hospitalization, she started a program of respiratory psychokinesis therapy and the following chest X-rays documented a gradually improving lung expansion. The drainages were progressively removed, and the patient was discharged 20 days after the operation. We managed to reduce the analgesic therapy, from Oxycodone 50 mg/bid to Acetaminophen 1 g/tid and Duloxetine 30 mg/die.

The histological analyses of the specimen revealed a pleomorphic undifferentiated G3 sarcoma, infiltrating the dermis and the skin, the underlying muscles, and the cortical layer of the sternum; the skin resection margins were all disease free (T2b N1 M0, G3, Stage III, according to the American Joint Committee on Cancer, 7th edition staging system for soft tissue sarcoma).

One month after the operation, a total body CT scan documented only the presence of little (diameter <1 cm) round-shaped mediastinal, left later cervical, and right axillary lymph nodes (Figure 1C).

The case was again discussed at a multidisciplinary meeting and an indication was given to perform Radiotherapy (RT) on the surgical site. The patient however refused it. A close surgical follow-up program was then established.

Three months after the surgery, the patient came to our attention complaining of an increased chest pain that required the reintroduction of opioids (Oxycodone 20 mg/bid).

Clinically the patient presented with skin ulceration above the inferior portion of the sternum and the insertion point of the lower titanium bar was exposed. There were no signs of infection.

The flap was vital, and the patient had no fever or respiratory symptoms (Figure 1D).

After an evaluation by our consulting plastic surgeon, the patient was scheduled for surgical repair of the defect through a mio-lipo-cutaneous flap using the contralateral breast.

Broad-spectrum antibiotic prophylaxis was started, and the patient was discharged the next day.

## Discussion

The RIS of the chest wall is commonly linked to a previous history of breast cancer or non-Hodgkin lymphoma treated with radiotherapy (1). Radio-associated sarcoma arises 10–20 years after the treatment, usually at the edge of the previous field of irradiation.

Radio-associated sarcoma is more aggressive than sporadic ones and they are often undifferentiated and high grade. Surgery, if feasible, still represents the best treatment option. To be radical, at least a 4 cm margin of the normal surrounding tissue should be excised. For lateral chest wall tumors, the free margins of the resection are one above the normal rib and one below (2). Chapelier et al. recommend stabilization of the chest wall only in patients with large anterior and anterolateral defects, especially after total sternotomy (3).

High-dose adjuvant radiotherapy should be avoided because of the prior exposure and the risk of complications such as necrosis of the underlying structures (bones, flaps, and grafts used to reconstruct the chest wall). Low dose, hypofractionated RT or brachytherapy remains to be feasible options, especially if the surgical margins are not clear. Adjuvant chemotherapy has not still produced convincing data (2).

Our surgical approach was focused on both demolition and reconstruction. The demolition involved a wide excision of the mass, ribs (II-VI), inferior part of the sternum, and pectoralis muscles. The most important aspect of the reconstruction developed around the reconstruction of the thoracic wall, using (layer by layer) acellular porcine dermis, titanium bars, acellular porcine dermis, latissimus dorsi flap, and skin grafts.

Marulli et al. used a similar approach in the case of giant chondrosarcoma arising from the right anterolateral chest, using titanium plates, dual-mesh patches, omental flap, pectoralis major, and latissimus dorsi muscle flaps (4). In both cases, using titanium bars or plates could provide stability to the chest cage and could allow a more anatomical and functional recreation of the chest wall.

When approaching a surgical procedure involving the excision of multiple ribs, part of the sternum, thoracic wall muscles, and the opening of the pleural cavity, the risk for post-op respiratory complications must be considered. An accurate pre-op respiratory function test must be carried out to define the surgical plan and the invasive ventilation modalities during the operation. A post-op respiratory rehabilitation program must be considered and started as soon as possible. Spicer et al., inspired by their findings and a literature review, concluded that the type of chest wall reconstruction is not associated with a significant impact on pulmonary or infectious wound complications after chest wall resection, rather, the number of resected ribs, subsequent chest wall defect size, and associated lung parenchymal resection determine the pulmonary morbidity after chest wall resection. We, therefore, opted for a tailored and individualized surgical approach (5).

Our patient was shortly extubated after the procedure and was then able to breathe spontaneously without oxygen support. No pulmonary complications occurred after the readmission in the ward, a broaden-spectrum antibiotic prophylaxis was carried on till the discharge. After an evaluation by our consulting pneumologist, the patient started a three-times-a-week respiratory rehabilitation.

A multidisciplinary team including oncologic, thoracic, and plastic surgeons, oncologists, radiotherapists, and radiologists is fundamental in the management of aggressive neoplasms such as sarcomas, which often require high-demolitive surgery and a very close post-procedural follow-up.

The management of post-operative pain is also relevant, especially when prosthetic implants like titanium bars are needed. Before the surgery, despite the heavy analgesic therapy, the quality of life of the patient was hardly impaired due to the chronic pain related to the mass. Post-operative acute pain required opioids and non-steroidal anti-inflammatory drugs (NSAID) again, but we were able to gradually reduce them over the next few weeks. At the discharge, the post-operative pain was well-controlled with Acetaminophen 1 g/tid. Notwithstanding, in the following weeks, the patient lamented the unpleasant and constant feeling of thoracic constriction that she described as the feeling of “being tied with a rope.” After an evaluation by the palliative care service that attributed the symptom to the titanium bars, a combination of opioids and serotonin-norepinephrine reuptake inhibitor (SNRI) was started with partial relief.

Unfortunately, 3 months after the operation, the bars ulcerated a little portion of the skin graft above the lower part of the sternum and a second operation was then mandatory, to stop the broadening of the ulceration and minimize the risk of infection.

We scheduled a regular follow-up every 3 months, due to the aggressiveness of the disease. Unfortunately, the patient relapsed locally and developed an even more locally advanced disease, for which she died 18 months after the diagnosis.

## Data Availability Statement

The original contributions presented in the study are included in the article/supplementary material, further inquiries can be directed to the corresponding author.

## Ethics Statement

Ethical review and approval was not required for the study on human participants in accordance with the local legislation and institutional requirements. The patients/participants provided their written informed consent to participate in this study.

## Author Contributions

CD, RM, and MR wrote the report. PD prepared the figures and reviewed the report. MS provided the pathology images and the interpretation. ST, EG, AZ, VV, AB, and SM supervised and approved the final version of the report. All authors contributed to the article and approved the submitted version.

## Conflict of Interest

The authors declare that the research was conducted in the absence of any commercial or financial relationships that could be construed as a potential conflict of interest.

## Publisher's Note

All claims expressed in this article are solely those of the authors and do not necessarily represent those of their affiliated organizations, or those of the publisher, the editors and the reviewers. Any product that may be evaluated in this article, or claim that may be made by its manufacturer, is not guaranteed or endorsed by the publisher.

## Abbreviations

RIS: radiation-induced sarcoma
FISH: fluorescence *in situ* hybridization
NSAID: non-steroidal anti-inflammatory drugs
SNRI: serotonin-norepinephrine reuptake inhibitor
ICU: intensive care unit.
